# The Pulmonary Artery Pulsatility Index Provides No Additional Prognostic Information in Pediatric Pulmonary Arterial Hypertension

**DOI:** 10.3390/children11101152

**Published:** 2024-09-24

**Authors:** Faye E. Smits, Chantal Lokhorst, Marlies G. Haarman, Mark-Jan Ploegstra, Rolf M. F. Berger, Johannes M. Douwes

**Affiliations:** Center for Congenital Heart Diseases, Department of Paediatric Cardiology, Beatrix Children’s Hospital, University Medical Center Groningen, University of Groningen, 9713 GZ Groningen, The Netherlands

**Keywords:** pulmonary arterial hypertension, children, survival, pulmonary hypertension

## Abstract

Background/Objectives: The pulmonary artery pulsatility index (PAPi, calculated as (SPAP − DPAP)/mRAP) has been suggested as a measure of right ventricular–vascular coupling (RVVC) and as a prognostic parameter in cardiovascular conditions, particularly right ventricular failure. This retrospective study investigated the relationship between the PAPi and its components with disease severity parameters, the RVVC, and clinical outcomes in children with pulmonary arterial hypertension (PAH). Methods: We analyzed data from 111 children from the Dutch National Registry with PAH. The PAPi (median 6.0 [3.9–8.3]) was calculated from heart catheterization data and the RVVC was determined as the TAPSE/sPAP ratio via echocardiography (0.25 ± 0.12 mm/mmHg). Disease severity was characterized by clinical, hemodynamic, and laboratory data. Cox proportional hazard modeling assessed the PAPi’s predictive value for transplant-free survival. Results: There was no correlation between the RVVC and PAPi (R = −0.208, *p* = 0.111, n = 60). The PAPi correlated negatively with uric acid (R = −0.387, *p* < 0.001) but not with other disease severity parameters. Mean right atrial pressure correlated with multiple disease severity indicators. Transplant-free survival rates at 1, 3, and 5 years were 87%, 79%, and 73%, respectively. Neither the PAPi nor its components correlated with transplant-free survival. Conclusions: In conclusion, the PAPi does not correlate with the RVVC and this study could not demonstrate any prognostic value of the PAPi regarding disease severity or outcomes in children with PAH, challenging its utility in this population.

## 1. Introduction

Pulmonary arterial hypertension (PAH) is a progressive disease characterized by pulmonary vascular remodeling [1]. PAH is defined by hemodynamic criteria, specifically as a mean pulmonary arterial pressure (mPAP) greater than 20 mmHg, a pulmonary arterial wedge pressure (PAWP) less than 15 mmHg, and a pulmonary vascular resistance index (PVRI) greater than 3 WU × m^2^ [2]. It affects approximately 15 to 20 per million people globally, with a higher prevalence in women and a notable incidence in pediatric populations [3]. PAH is associated with significant social and economic burdens, including frequent hospitalizations, reduced quality of life, and substantial healthcare costs [4]. The pulmonary vascular changes in PAH include typical neo-intimal lesions in distal pulmonary arterioles and stiffening of the more proximal pulmonary vascular tree, including the conduit vessels [5]. This pulmonary vascular remodeling leads to an increased right ventricular (RV) afterload [1]. To maintain cardiac output, the right ventricle adapts to the increase in afterload by enhancing contractility and wall thickening (ventricular–vascular coupling) [6]. Eventually, the afterload exceeds the RV-adaptive mechanisms, and RV dilation occurs [1]. In the advanced stages of the disease, RV failure occurs, eventually leading to the patient’s death. Untreated, the median survival is 2.5 years and even with treatment the prognosis is poor [7]. Therefore, PAH in children is still associated with significant morbidity and mortality.

The pulmonary artery pulsatility index (PAPi) has recently been suggested as a measure of right ventricular–vascular coupling (RVVC), allowing for clinical risk stratification in adults with PAH [4]. The PAPi is defined as [systolic pulmonary arterial pressure (SPAP) minus diastolic pulmonary arterial pressure (DPAP)] divided by mean right atrial pressure (mRAP) [8]. While PAPi has been suggested to be an independent predictor of survival in adults with PAH and for RV failure in patients with acute inferior myocardial infarction and after left ventricular (LV) assist device implantation [9], its prognostic value has been debated. Right ventriculo-arterial uncoupling, a condition where RV function and the afterload are mismatched, has been associated with RV dysfunction and dilation and worse outcomes in PAH [3,5,10]. However, the PAPi is influenced by multiple parameters, including pulmonary arterial capacitance, stroke volume, right atrial pressure and pulmonary arterial wedge pressure, and in PAH it may primarily reflect the right ventricular afterload, represented by the right ventricular time constant [5], rather than the RVVC (the ratio of right ventricular end-systolic elastance to effective pulmonary arterial elastance) [10].

No studies have specifically examined the PAPi’s role in children with PAH. Given that hemodynamic characteristics and RV adaptation in children differ from those in adults, we aimed to investigate the clinical value of the PAPi in this population. We therefore investigated the relationship between the PAPi and RVVC, established markers of disease severity, and furthermore, the prognostic value of the PAPi and its additional value compared to its single components, mRAP and pulse pressure (PP), in children with different etiologies of PAH.

Research Question: Is the PAPi a predictor of disease severity and outcomes in children with PAH?

## 2. Materials and Methods

### 2.1. Study Design and Population

This study is a retrospective analysis of data from the prospective, longitudinal Dutch National Registry for Pulmonary Hypertension in childhood. In the Netherlands, all pediatric patients with suspected PAH are referred to the National Referral Center for Pediatric Pulmonary Hypertension at the University Medical Center Groningen. These patients undergo a comprehensive diagnostic process, including confirmation of the diagnosis through cardiac catheterization [11]. Treatment follows international guidelines adapted for children, with regular follow-up visits every 3–12 months to monitor clinical endpoints [11]. Data from these evaluations and treatments are recorded in the Dutch pediatric PAH registry, maintained by a dedicated coordinator with automated quality checks. The study protocol complies with the ethical standards of the 1975 Declaration of Helsinki, as evidenced by prior approval from the institution’s human research committee, and written informed consent was obtained from the patients or their caregivers [11].

For this study, all consecutive children with PAH confirmed by heart catheterization were identified in the Dutch National registry. These patients were diagnosed between the years 1993 and 2023. The diagnosis was confirmed by cardiac catheterization, following the criteria proposed by the latest WSPH in 2018 (mean right atrial pressure (mPAP) > 20 mmHg, pulmonary atrial wedge pressure (PAWP) < 15 mmHg and a pulmonary vascular resistance index (PVRI) > 3 WU × m^2^ ) [2]. Patients were excluded if the PAPi could not be calculated due to missing values. The PAPi was calculated using the formula (SPAP − DPAP)/mRAP, measured during the diagnostic heart catherization (HC). Over time, patients were managed according to contemporary treatment recommendations.

Disease severity was assessed by established invasive and non-invasive variables, including clinical, hemodynamic and laboratory metrics [5]. In the Dutch National registry WHO functional class (WHO-FC), six-minute walking distance (6MWD) and laboratory tests are collected at the time of the HC and at standardized time points during follow up. Hemodynamic data derived from the HC included the cardiac index (CI), PVRI, mPAP, mRAP, and PP, laboratory tests included N-terminal pro-B-type natriuretic peptide (NT-proBNP) and uric acid.

The outcome measurements of this study were transplant-free survival at 1-, 3- and 5-years and disease severity.

### 2.2. Echocardiographic Methods

To investigate whether the PAPi is related to the right ventricular to pulmonary vascular coupling, the correlation of the PAPi with the TAPSE/sPAP ratio was analyzed. The TAPSE/sPAP ratio is a validated and widely accepted noninvasive measurement of RV—arterial coupling in patients with pulmonary hypertension [10]. TAPSE was obtained from an echocardiography preformed within six months of heart catheterization. The sPAP was estimated based on the tricuspid regurgitation doppler maximal flow velocity (TAPSE/sPAP non-invasive). Since not all patients exhibited tricuspid regurgitation, a secondary analysis was conducted, calculating the TAPSE/sPAP ratio using the sPAP from the heart catheterization (TAPSE/sPAP invasive).

### 2.3. Statistical Analysis

Patients were assigned to four subgroups according to PAH etiology: heritable or idiopathic PAH (HPAH/IPAH), PAH associated with congenital heart disease (PAH-CHD) with post-tricuspid shunt, PAH-CHD with no post-tricuspid shunt, and finally “mixed etiologies of PAH”.

Baseline variables were tested for normality of distribution using Q-Q plots. Continuous variables that were normally distributed were displayed as mean and standard deviation, while non-normally distributed data were presented as median and interquartile range. Categorical variables were reported as absolute numbers and percentages. One-way ANOVA was used to examine intergroup differences for normally distributed data and the Kruskal–Wallis test was applied for non-normally distributed data. The chi-square test was used for categorical variables. Additionally, the Bonferroni adjustment (post hoc analysis) was applied to assess significant differences between subgroups of categorical variables, namely WHO-FC and sex.

The correlations between the PAPi, mRAP and PP with disease severity variables was also analyzed. Spearman’s correlation coefficient was used for continuous non-normally distributed variables.

To determine whether the PAPi, mRAP and PP were significant predictors of clinical outcomes, survival analysis models were conducted at 1-, 3 and 5-years. The endpoints were mortality and lung transplantation. Patients without an endpoint were censored at their last follow-up visit. Cox regression analysis was used to examine the association between multiple independent variables and outcome. A multivariate Cox regression analysis was performed to correct these correlations for three other clinically relevant and potentially interfering variables: PVRI, cardiac index, and diagnosis. The estimates are presented as hazard ratios with 95% confidence intervals. Kaplan–Meier graphs were used to illustrate the estimated survival. A *p*-value of less than 0.05 was considered statistically significant. All statistical analyses were conducted using IBM SPSS Statistics 28.

## 3. Results

### 3.1. Study Population

A total of 126 PAH patients diagnosed between 1993 and 2023 were identified in the Dutch National Registry. Of these, two patients were excluded due to diagnoses of significant pulmonary hypertension associated comorbidities that might have biased the analyses (one patient with pulmonary venous obstruction and unilateral PAH in only one lung, and one patient with severe respiratory disease, right sided pneumonectomy, and pulmonary venous hypertension). Thirteen additional patients were excluded due to incomplete hemodynamic data, which prevented the calculation of PAPi. This left a study population of 111 patients, of whom 57% were female. The median age was 7.2 (2.2–13.2) years. The majority of the cohort, 67 patients (63%), had advanced disease at diagnosis, characterized by WHO-FC of III or IV. Baseline characteristics are shown in Table 1.

Among the 111 children, 45 children (41%) had HPAH/IPAH, 25 children (22%) PAH-CHD with post-tricuspid shunt, 28 children (25%) PAH-CHD without post-tricuspid shunt, and 13 children (12%) had mixed etiologies of PAH. The latter group included seven patients with pulmonary veno-occlusive disease (PVOD), one additional patient with PVOD and concomitant pulmonary arteriovenous malformation (PAVM), three patients with portopulmonary hypertension (associated with Alagille syndrome, cystic fibrosis and portal thrombosis resp.), one patient with connective tissue disease, and finally, one patient with Kleefstra syndrome, VSD, and mild mitral valve stenosis associated with combined pre- and post-capillary pulmonary hypertension.

**Figure 1:**
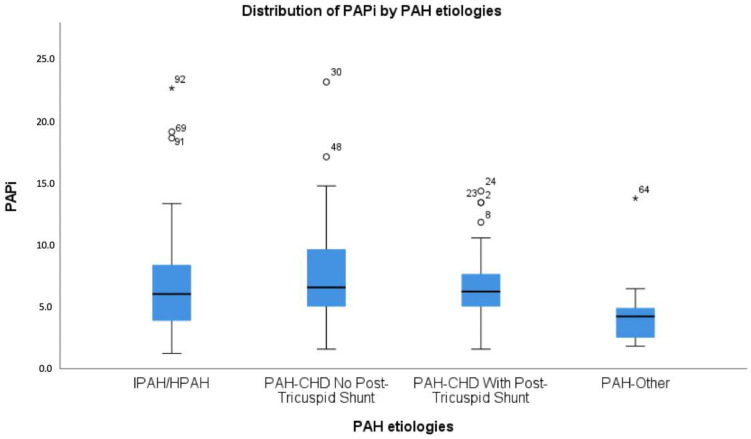
Boxplot of the median PAPi within the different etiological PAH subgroups. Outliers are marked with small circles for values that are more than 1.5 times the IQR below the first quartile or above the third quartile, and stars for ‘far out’ (extreme) values that are more than 3 times the IQR beyond the first or third quartile.

Patients with PAH-CHD and post-tricuspid shunt were significantly younger at baseline (4.71 [0.69–11.75] years) and were more often female (80%, *p* = 0.004) compared to the other subgroups. No statistically significant differences between subgroups were found regarding conventional hemodynamic variables. Within the total patient population, the PAPi was not normally distributed with a median value of 6.0 (4.0–8.0). The PAPi differed between the subgroups (*p* = 0.032): In children with PAH-CHD without shunt, the PAPi was the highest (6.5 [5.0–9.8]), whereas it was the lowest in the PAH-mixed group (4.2 [2.5–5.3]). The distribution of the PAPi within the four subgroups is shown in Figure 1.

### 3.2. Coupling

In 77 patients, an echocardiography was performed within a 6-month time frame of the heart catheterization. In 60 of these patients, tricuspid regurgitation was present. For these 60 patients, the fully non-invasively measured TAPSE/sPAP ratio was 0.24 ± 0.11 and did not correlate with PAPi (R = −0.208, *p* = 0.111). In a secondary analysis, the TAPSE/sPAP ratio was calculated using the sPAP that was invasively measured during heart catheterization (n = 77). For these patients the TAPSP/sPAP ratio was 0.25 ± 0.12 and did not correlate with PAPi (R = −0.116, *p* = 0.316). Neither the invasively and non-invasively measured TAPSE/sPAP ratios correlated with transplant-free survival at 1, 3, or 5 years.

### 3.3. Disease Severity Variables

In the overall study population, PAPi correlated negatively with uric acid (n = 76; R = −0.387; *p* < 0.001). No correlations could be demonstrated between PAPi and WHO-FC, 6MWD, NT-proBNP, or CI. mRAP correlated significantly with WHO-FC (n = 107; R = 0.224, *p* = 0.020), NT-proBNP (n = 81; R = 0.292; *p* = 0.008), uric acid (n = 76; R = 0.41; *p* < 0.001), and mPAP (n = 111; R = 0.23; *p* = 0.017). PP correlated with mPAP (n = 111; R = 0.510; *p* < 0.001) and PVRI (n = 111; R = 0.380; *p* < 0.001) (Table 2).

Analyses conducted separately within each of the four subgroups showed similar results. The results for the IPAH/HPAH subgroup are shown in Table 3. In this subgroup, the PAPi correlated significantly with uric acid (n = 32, R = −0.523, *p* = 0.002) and CI (n = 44, R = 0.374, *p* = 0.012); however, these associations appeared driven by the strong association of both these parameters with the mRAP (Table 3). Additionally, in this subgroup, the mRAP was associated with other invasive hemodynamic parameters of disease severity, such as the PVRI and mPAP.

### 3.4. Survival Analysis

**Figure 2:**
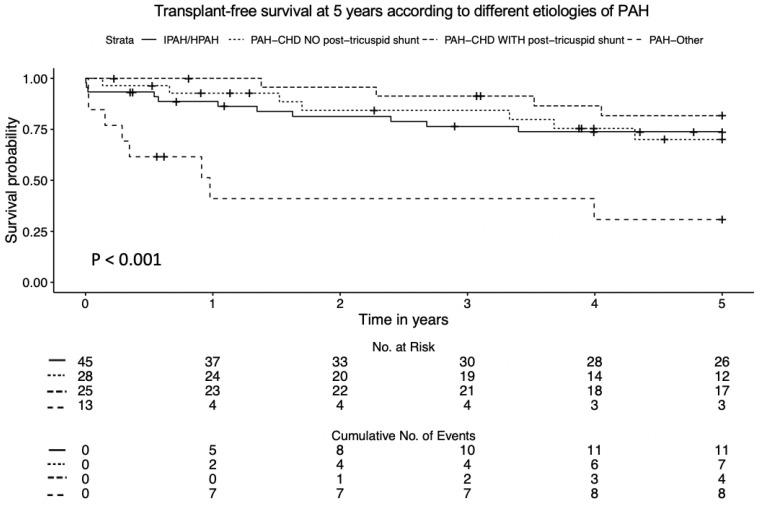
Kaplan–Meier survival curve for different etiologies.

In the entire study group, the 1-, 3-, and 5-year transplant-free survival rates were 87%, 79%, and 73%, respectively. The PAH-mixed etiologies group demonstrated the lowest transplant-free survival rates over time, with 1-, 3-, and 5-year survival rates of 46%, 46%, and 39%, respectively. In contrast, children with CHD with a post-tricuspid shunt showed the highest probability of transplant-free survival (log-rank *p* < 0.001) (Figure 2).

Cox regression analyses, conducted both in the total study group and the separate IPAH/HPAH subgroup, did not demonstrate a relationship between the PAPi and 1-, 3- or 5-year transplant-free survival. Similarly, its individual components, mRAP and PP, were not associated with transplant-free survival (Table 4 and Table 5). In the multivariate Cox regression analysis, the univariate analyses were corrected for the PVRI, cardiac index, and diagnosis. No correlation was found between transplant-free survival and the PAPi, mRAP, or PP when adjusted for the PVRI, cardiac index, and diagnosis (Table A1).

## 4. Discussion

In this study, we investigated the correlation between the PAPi, RVVC, and clinical disease severity in children with PAH, as well as the prognostic value of the PAPi for transplant-free survival. Although the PAPi has been a suggested measure for ventricular–vascular coupling and a predictor of outcomes in adult patients with cardiovascular conditions, including PAH, our current study showed no correlation of the PAPi with RVVC or any prognostic value in children with PAH.

Our results show that the PAPi does not correlate with transplant-free survival in the pediatric PAH population. These findings remained consistent after adjusting the analyses for known outcome predictors that could potentially interfere with the correlation between the PAPi and transplant-free survival. This contrasts with findings in adults with PAH, where studies have shown the PAPi to be predictive of outcomes, with a lower PAPi associated with 1-year mortality [8,10]. However, even in adult patients, controversy exists regarding the additional value of this composite variable, as it is suggested to be primarily driven by its component mRAP, a well-known predictor of RV failure and death [12].

Children with PAH are known to preserve RV function and cardiac output longer than adults, even in the presence of advanced pulmonary vascular disease [13]. An increase in the mRAP occurs only with the onset of RV failure and is a rather late event in pediatric PAH, typically not seen until end-stage disease. In children with IPAH/HPAH, we found the correlations between the mRAP and CI and the mRAP and PVRI to be much stronger than those with the PAPi, supporting the idea that the PAPi’s predictive value is not reflective of VVC but is primarily driven by the mRAP, a marker of RV maladaptation and failure.

TAPSE/sPAP is a well-established measurement of RV-to-pulmonary vascular coupling. The PAPi has been suggested as a measurement of RV-to-pulmonary vascular coupling. Multiple studies have demonstrated that a low TAPSE/sPAP ratio indicates abnormal RV–vascular coupling [10]. Kazimierczyk et al. showed that patients with a lower TAPSE/sPAP had a worse prognosis, with a ratio ≤ 0.32 mm/mmHg being a significant predictor of all-cause mortality [14,15]. Furthermore, the 2022 ESC/ERS Guidelines for pulmonary hypertension include TAPSE/sPAP as a key echocardiographic indicator of RV coupling in PAH [16]. In our cohort, no correlation between the PAPi and TAPSE/sPAP was observed, suggesting that the PAPi may not reliably represent RV-to-pulmonary vascular coupling in this population.

The etiology of PAH is known to differ between adults and children. In pediatric PAH, associated CHD is more frequent compared to adults, whereas adults often have PAH linked to connective tissue disease [17,18]. In the current study, the PAH-mixed etiologies group had the highest mRAP and lowest PAPi (4.2 [2.5–5.3]) and the worst outcome, with the lowest chance of survival at 5 years (39%) compared with the other three groups that had a lower mRAP and higher PAPi. Seven patients in the mixed-etiologies group were diagnosed with PVOD, a rapidly progressive pulmonary vascular disease associated with early RV failure and death [19]. Almost one-quarter (23%) of the children in the current study had PAH-CHD with a post-tricuspid shunt. These patients were characterized by a younger age at diagnosis, a higher CI and a higher PP. The circulatory physiology of these patients differs from the other subgroups due to the presence of a post-tricuspid shunt, which may form a pop-off and thereby a relief of the RV [20,21]. Consequently, pulmonary hemodynamics are affected by such a shunt. Together, this may explain the better survival in these patients, associated with a low mRAP and preserved RV function for a relatively long time. In this context, the PAPi or other indirect VVC-metrics cannot be extrapolated from patients without shunts to patients with post-tricuspid shunts.

We found a correlation between both the mRAP and PAPi with uric acid levels. Higher serum levels of uric acid have previously been shown to correlate with PAH severity and worsening [22]. In adults, serum levels of uric acid may be affected by common comorbidities, such as hypertension and cardiovascular and renal conditions, limiting its prognostic value in adults with PAH. However, in children with PAH, uric acid levels have been shown to correlate with invasive hemodynamics, even in those with PAH-CHD and shunts, and serial measurements have been predictive of outcomes [23,24]. The mechanism behind it is not fully understood but enhanced arginase activity leading to decreased nitric oxide availability in endothelial cells and inhibition of acetylcholine-mediated vasodilatation, will affect the pulmonary vasculature and contribute to disease progression [25,26]. Furthermore, uric acid is a product of purine metabolism and its levels are influenced by factors such as cellular turnover, hypoxia, and inflammation—all of which are prominent in PAH. The study by Watanabe et al. found that elevated lung uric acid levels are directly associated with worsening pulmonary arterial hypertension (PAH), as they contribute to vascular remodeling and increased right ventricular pressure. In PAH, increased mRAP reflects a worsening of right ventricular function due to the elevated pressures in the pulmonary circulation, explaining its correlation with uric acid.

The PAPi did not correlate with the other well-established variables of disease severity in pediatric PAH, such as the WHO-FC, mPAP, PVRi, or NT-proBNP. In contrast to the PAPi, the mRAP did show a correlation with the WHO-FC and NT-proBNP. The NT-proBNP is a well-established cardiac marker for cardiovascular disease including PAH. Higher levels of NT-proBNP are indicative of RV dysfunction and hypertrophy, which is seen as PAH progresses [27]. Furthermore, a higher WHO-FC, a strong marker for disease severity in PAH, was seen in patients with a higher mRAP.

In this pediatric PAH cohort, the PAPi did not provide prognostic information independent from other well-established mono-component clinical, laboratory, or hemodynamic risk factors. We could demonstrate only a limited association with disease severity parameters but not with outcome. It seems that the limited associations we found were largely driven by the mRAP, which is known to be a good prognostic parameter in children and adults with PAH [7].

### Strengths and Limitations

This study had several strengths. First, it is a large, well and consistently maintained study population for the rare disease of pediatric PAH. In this study, the HCs were only performed in the UMCG by skilled pediatric cardiologists to limit inter- and intra-user variability. However, this study had some limitations. One limitation was the small population size within individual subgroups, resulting in a low number of adverse events. Another limitation was that the RVVC (TAPSE/sPAP) data were available for only a subset of the population (77 invasively and 60 non-invasively out of 111 patients), which may affect the generalizability of the findings. Additionally, the overall population included heterogenous PAH diagnoses; to overcome this, we also investigated the IPAH/HPAH group separately. Lastly, the PAPi is likely to reflect right ventricular afterload, which has been shown to be influenced by heart rate [28]. As our study population ranged from 2.2 to 13.2 years, age-related variations in heart rate may play a role in our analysis.

## 5. Conclusions

In this study, we were unable to demonstrate a correlation between the PAPi and ventricular-vascular coupling or outcomes. The limited correlations with established parameters of disease severity seemed to be driven predominantly by the mRAP. To conclude, in pediatric PAH, the composite variable PAPi provided no incremental predictive value to the monocomponent variable mRAP regarding outcomes and disease severity.

## Figures and Tables

**Table 1:** Baseline characteristic according to children with different etiologies of PAH.

Variables	All (n = 111)	HPAH/IPAH (n = 45)	CHD without Post-shunt (n = 28)	CHD with Post-shunt (n = 25)	Mixed Etiologies (n = 13)	*p*-Value
Age, at baseline (years)	7.2 (2.2–13.2)	8.0 (4.5–14.1)	6.1 (1.3–12.8)	4.7 (0.7–11.8)	8.2 (6.3–12.6)	0.090
Sex (female) n (%)	63 (57)	20 (44)	19 (68)	20 (80)	4 (31)	**0.004**
WHO-FC						
I–II, n (%) n = 40	40 (37)	13 (30)	14 (52)	10 (42)	3 (23)	0.197
III–IV, n (%) n = 67	67 (63)	30 (70)	13 (48)	14 (58)	10 (67)	
NT-proBNP (ng/L) n = 81	497 (140–1522)	774 (179–2308)	314 (88–1038)	541 (117–1368)	182 (115–1551)	0.599
Uric acid (mmol/L) n = 76	0.31 ± 0.11	0.31 ± 0.12	0.29 ± 0.08	0.32 ± 0.12	0.31 ± 0.11	0.678
6MWD (meters) n = 51	356 (306–392)	372 (320–416)	372 (328–475)	312 (264–376)	368 (288–378)	0.096
Cardiac index (L/min/m^2^) n = 109	3.4 ± 1.7	3.1 ± 1.2	3.0 ± 0.7	4.0 ± 3.1	3.6 ± 1.1	0.133
mPAP (mmHg) n = 111	50 ± 18	51 ± 20	48 ± 17	54 ± 17	45 ± 16	0.448
mRAP (mmHg) n = 111	6 (4–8)	6 (4–8)	5 (4–7)	6 (4–8)	8 (5–10)	0.496
PP (mmHg) n = 111	35 (28–44)	34 (30–44)	35 (27–46)	37 (31–42)	28 (23–37)	0.131
PVRI (WU × m^2^ ) n = 111	12 (7–23)	12 (7–25)	14 (6–23)	14 (9–19)	8 (6–15)	0.522
PAPi n = 111	6.0 (3.9–8.3)	6.0 (3.9–8.5)	6.5 (5.0–9.8)	6.2 (4.7–8.2)	4.2 (2.5–5.3)	**0.032**
TAPSE (mm) invasive n = 90	15.86 ± 3.53	15.88 ± 3.04	15.50 ± 4.37	14.11 ± 2.54	16.79 ± 3.04	0.216
TAPSE/sPAP (mm/mmHg) invasive n = 77	0.25 ± 0.12	0.25 ± 0.10	0.24 ± 0.13	0.20 ± 0.06	0.31 ± 0.15	0.124
Time HC to Coupling invasive (months) n = 96	0.03 (0.26–1.39)	0.03 (0.26–0.18)	0.00 (0.39–4.04)	1.45 (0.26–4.67)	0.12 (0.22–0.22)	0.538
TAPSE (mm) non-invasive n = 90	15.6 ± 3.52	15.83 ± 2.96	15.50 ± 4.13	14.55 ± 3.01	15.83 ± 2.96	0.501
TAPSE/sPAP (mm/mmHg) non-invasive n = 60	0.24 ± 0.11	0.24 ± 0.10	0.21 ± 0.10	0.22 ± 0.07	0.31 ± 0.15	0.121
Time HC to Coupling non-invasive (months)	0.02 (0.26–1.88)	0.03 (0.26–1.10)	0.00 (0.39–4.04)	1.91 (0.26–4.67)	0.10 (0.22–0.28)	0.679

Continuous data are shown as median (interquartile range) (normally distributed) and mean (standard deviation) (non-normally distributed). Categorical data are shown as absolute numbers (%). One-way ANOVA was used for normally distributed data, and the Kruskal-Wallis test for non-normal data. Categorical data were analyzed with the chi-square test, and significant differences between subgroups were assessed using the Bonferroni adjustment. WHO-FC, World Health Organization functional class; 6MWD, six-minute walk test; mPAP, mean pulmonary artery pressure; mRAP, mean right atrial pressure; PP, pulse pressure; PVRI, pulmonary vascular resistance index; PAPi, pulmonary artery pulsatility index; WU × m^2^, Wood’s Units times square meter; TAPSE, tricuspid annular plane systolic excursion; TAPSE/sPAP, tricuspid annular plane systolic excursion/systolic pulmonary artery pressure ratio; Time HCtoCoupling, time between heart catherization and echocardiogram in months.

**Table 2:** Correlations between PAPi, mRAP, and PP with disease severity variables, total group.

	PAPi (n = 111)		mRAP (n = 111)		PP (n = 111)	
Variables	R	*p*-Value	R	*p*-Value	R	*p*-Value
Age, at baseline (years) n = 111	−0.09	0.366	0.06	0.515	0.01	0.953
Sex n = 111	0.11	0.257	−0.07	0.440	0.12	0.217
WHO I-II-III-IV n = 107	−0.18	0.067	0.22	**0.020**	−0.01	0.924
NT-proBNP (ng/L) n = 81	−0.12	0.279	0.29	**0.008**	0.1.	0.333
Uric acid (mmol/L) n = 76	−0.39	**<0.001**	0.41	**<0.001**	−0.10	0.398
6MWD (meters) n = 51	0.25	0.08	−0.20	0.162	0.17	0.245
Cardiac index (L/min/m^2^) n = 109	0.14	0.150	−0.19	0.05	−0.043	0.66
mPAP (mmHg) n = 111	0.12	0.201	0.23	**0.017**	0.51	**<0.001**
PVRI (WU × m^2^) n = 111	0.08	0.412	0.18	0.054	0.38	**<0.001**

The correlation was assessed using Spearman’s correlation coefficient for continuous, non-normally distributed variables. R, Spearman’s correlation coefficient; WHO-FC, World Health Organization functional class; 6MWD, six-minute walk test; mPAP, mean pulmonary artery pressure; mRAP, mean right atrial pressure; PP, pulse pressure; PVRI, pulmonary vascular resistance index; PAPi, pulmonary artery pulsatility index.

**Table 3:** Correlations between PAPi, mRAP, and PP with disease severity variables for the IPAH/HPAH group.

	PAPi (n = 45)		mRAP (n = 45)		PP (n = 45)	
Variables	R	*p*-Value	R	*p*-Value	R	*p*-Value
Age, at baseline (years) n = 45	−0.17	0.256	0.15	0.312	−0.03	0.857
Sex n = 45	0.05	0.745	−0.05	0.769	0.06	0.702
WHO I-II-III-IV n = 43	−0.15	0.326	0.26	0.097	0.12	0.454
WHO I/II vs. III/IV n = 43	−0.03	0.846	0.21	0.169	**0.30**	**0.047**
NT-proBNP (ng/L) n = 34	−0.13	0.480	0.27	0.130	0.16	0.382
Uric acid (mmol/L) n = 32	**−0.52**	**0.002**	**0.55**	**0.001**	−0.11	0.544
6MWD (meters) n = 23	0.29	0.177	−0.18	0.424	0.18	0.404
Cardiac index (L/min/m^2^) n = 44	**0.37**	**0.012**	**−0.45**	**0.002**	−0.08	0.591
mPAP (mmHg) n = 45	0.01	0.936	**0.33**	**0.026**	**0.57**	**<0.001**
PVRI (WU × m^2^) n = 45	−0.12	0.426	**0.38**	**0.009**	**0.44**	**0.002**

The correlation was assessed using Spearman’s correlation coefficient for continuous, non-normally distributed variables. R, Spearman’s correlation coefficient; WHO-FC, World Health Organization functional class; 6MWD, six-minute walk test; mPAP, mean pulmonary artery pressure; mRAP, mean right atrial pressure; PP, pulse pressure; PVRI, pulmonary vascular resistance index; PAPi, pulmonary artery pulsatility index.

**Table 4:** Cox regression for PAPi, mRAP, and PP predicting transplant-free survival for the total group.

	PAPi		mRAP		PP		
Years	HR (95% CI)	*p*-Value	HR (95% CI)	*p*-Value	HR (95% CI)	*p*-Value	No. of Events
**1**	0.916 (0.781–1.074)	0.281	1.038 (0.902–1.194)	0.605	0.993 (0.951–1.037)	0.750	14
**3**	0.927 (0.823–1.045)	0.214	1.051 (0.944–1.168)	0.364	0.982 (0.948–1.018)	0.326	23
**5**	0.938 (0.848–1.037)	0.214	1.059 (0.967–1.160)	0.216	0.992 (0.963–1.021)	0.574	30

Cox regression analysis was applied to assess the association between these independent variables and clinical outcomes. Endpoints were mortality and lung transplantation, with patients who did not reach an endpoint being censored at their last follow-up visit. PAPi, pulmonary artery pulsatility index; mRAP, mean right atrial pressure; PP, pulse pressure.

**Table 5:** Cox regression for PAPi, mRAP, and PP predicting transplant-free survival for the IPAH/HPAH group.

	PAPi		mRAP		PP		
Years	HR (95% CI)	*p*-Value	HR (95% CI)	*p*-Value	HR (95% CI)	*p*-Value	No. of Events
1	0.977 (0.792–1.206)	0.830	0.958 (0.746–1.229)	0.735	1.026 (0.970–1.085)	0.376	5
3	0.883 (0.724–1.078)	0.222	1.048 (0.917–1.198)	0.495	1.004 (0.957–1.052)	0.884	10
5	0.891 (0.740–1.072)	0.221	1.043 (0.916–1.187)	0.523	1.005 (0.961–1.051)	0.820	11

Cox regression analysis was applied to assess the association between these independent variables and clinical outcomes. Endpoints were mortality and lung transplantation, with patients who did not reach an endpoint being censored at their last follow-up visit. PAPi, pulmonary artery pulsatility index; mRAP, mean right atrial pressure; PP, pulse pressure.

## Data Availability

The data that support the findings of this study may be available from the corresponding author upon reasonable request due to privacy restrictions.

## Appendix A

**Table A1:** Multivariate Cox regression for PAPi, mRAP, and PP, corrected for PVRI, cardiac index, and diagnosis, predicting transplant-free survival for the total group.

	PAPi		mRAP		PP		
Years	HR (95% CI)	*p*-Value	HR (95% CI)	*p*-Value	HR (95% CI)	*p*-Value	No. of Events
**1**	1.022 (0.893–1.169)	0.753	0.853 (0.693–1.050)	0.133	1.014 (0.969–1.061)	0.554	14
**3**	0.986 (0.884–1.100)	0.803	0.975 (0.851–1.118)	0.720	0.996 (0.960–1.034)	0.845	23
**5**	0.996 (0.908–1.091)	0.925	0.963 (0.854–1.086)	0.539	1.001 (0.971–1.033)	0.932	30

Multivariate Cox regression analysis was applied to assess the association between independent variables and clinical outcomes, corrected for PVRI, cardiac index, and diagnosis. Endpoints were mortality and lung transplantation, with patients who did not reach an endpoint being censored at their last follow-up visit. PAPi, pulmonary artery pulsatility index; mRAP, mean right atrial pressure; PP, pulse pressure.

## References

[1] Vonk Noordegraaf A., Westerhof B.E., Westerhof N. (2017). The Relationship between the Right Ventricle and its Load in Pulmonary Hypertension. J. Am. Coll. Cardiol..

[2] Galiè N., McLaughlin V.V., Rubin L.J., Simonneau G. (2019). An overview of the 6th World Symposium on Pulmonary Hypertension. Eur. Respir. J..

[3] Galiè N., Corris P.A., Frost A., Girgis R.E., Granton J., Jing Z.C., Klepetko W., McGoon M.D., McLaughlin V.V., Preston I.R. (2013). Updated Treatment Algorithm of Pulmonary Arterial Hypertension. J. Am. Coll. Cardiol..

[4] McGoon M.D., Benza R.L., Escribano-Subias P., Jiang X., Miller D.P., Peacock A.J., Pepke-Zaba J., Pulido T., Rich S., Rosenkranz S. (2013). Pulmonary Arterial Hypertension: Epidemiology and Registries. J. Am. Coll. Cardiol..

[5] Humbert M., Kovacs G., Hoeper M.M., Badagliacca R., Berger R.M.F., Brida M., Carlsen J., Coats A.J.S., Escribano-Subias P., Ferrari P. (2022). 2022 ESC/ERS Guidelines for the diagnosis and treatment of pulmonary hypertension. Eur. Heart J..

[6] Dufva M.J., Ivy D., Campbell K., Lam A., Rauff A., Breeman K.T.N., Douwes J.M., Berger R.M.F., Kheyfets V.O., Hunter K. (2021). Ventricular–vascular coupling is predictive of adverse clinical outcome in paediatric pulmonary arterial hypertension. Open Heart.

[7] Ivy D. (2016). Pulmonary Hypertension in Children. Cardiol. Clin..

[8] Mazimba S., Welch T.S., Mwansa H., Breathett K.K., Kennedy J.L.W., Mihalek A.D., Harding W.C., Mysore M.M., Zhuo D.X., Bilchick K.C. (2019). Haemodynamically Derived Pulmonary Artery Pulsatility Index Predicts Mortality in Pulmonary Arterial Hypertension. Heart Lung Circ..

[9] Lim Y., Low T., Chan S.P., Lin W., Teo T.W., Jang J.J., Kuntjoro I., Tay E.L.-W., Yip J.W.-L. (2021). Does pulmonary artery pulsatility index predict mortality in pulmonary arterial hypertension?. ESC Heart Fail..

[10] Tello K., Wan J., Dalmer A., Vanderpool R., Ghofrani H.A., Naeije R., Roller F., Mohajerani E., Seeger W., Herberg U. (2019). Validation of the Tricuspid Annular Plane Systolic Excursion/Systolic Pulmonary Artery Pressure Ratio for the Assessment of Right Ventricular-Arterial Coupling in Severe Pulmonary Hypertension. Circ. Cardiovasc. Imaging.

[11] Douwes J.M., Berger R.M.F. (2017). Pediatric pulmonary arterial hypertension. Curr. Opin. Pulm. Med..

[12] Ostad S., Sugarman J., Alkhodair A., Liang J., Mielniczuk L.M., Hambly N., Helmersen D., Hirani N., Thakrar M., Varughese R. (2023). Association Between the Pulmonary Artery Pulsatility Index and Prognosis in Pulmonary Arterial Hypertension: A Multicentre Study. CJC Open.

[13] Berger R.M.F., Beghetti M., Humpl T., Raskob G.E., Ivy D.D., Jing Z.C., Bonnet D., Schulze-Neick I., Barst R.J. (2012). Clinical features of paediatric pulmonary hypertension: A registry study. Lancet.

[14] Kazimierczyk R., Kazimierczyk E., Knapp M., Sobkowicz B., Malek L.A., Blaszczak P., Ptaszynska-Kopczynska K., Grzywna R., Kaminski K.A. (2021). Echocardiographic Assessment of Right Ventricular–Arterial Coupling in Predicting Prognosis of Pulmonary Arterial Hypertension Patients. J. Clin. Med..

[15] Colalillo A., Hoffmann-Vold A.M., Pellicano C., Romaniello A., Gabrielli A., Hachulla E., Smith V., Simeón-Aznar C.-P., Castellví I., Airò P. (2023). The role of TAPSE/sPAP ratio in predicting pulmonary hypertension and mortality in the systemic sclerosis EUSTAR cohort. Autoimmun. Rev..

[16] Rosenzweig E.B., Abman S.H., Adatia I., Beghetti M., Bonnet D., Haworth S., Ivy D.D., Berger R.M. (2019). Paediatric pulmonary arterial hypertension: Updates on definition, classification, diagnostics and management. Eur. Respir. J..

[17] Barst R.J., Ivy D., Badesch D.B., Benza R.L., Elliott C.G., Farber H.W., Frost A., Krichman A., Liou T., Raskob G. (2009). 228: REVEAL Registry: Comparison of Patients with Childhood-Onset and Adult-Onset Idiopathic Pulmonary Arterial Hypertension. J. Heart Lung Transplant..

[18] Hajouli S., Moustafa M.A., Memoli J.S.W. (2019). Pulmonary Veno-Occlusive Disease: A Rare Cause of Pulmonary Hypertension. J. Investig. Med. High. Impact Case Rep..

[19] D’Alto M., Mahadevan V.S. (2012). Pulmonary arterial hypertension associated with congenital heart disease. Eur. Respir. Rev..

[20] Kozlik-Feldmann R., Hansmann G., Bonnet D., Schranz D., Apitz C., Michel-Behnke I. (2016). Pulmonary hypertension in children with congenital heart disease (PAH-CHD, PPHVD-CHD). Expert consensus statement on the diagnosis and treatment of paediatric pulmonary hypertension. The European Paediatric Pulmonary Vascular Disease Network, endorsed by ISHLT and DGPK. Heart.

[21] Seyyedi S.R., Malekmohammad M., Chitsazan M., Behzadnia N., Sadr M., Hashemian S.M., Sharif-Kashani B. (2017). Relationship between Serum Uric Acid Levels and the Severity of Pulmonary Hypertension. Tanaffos.

[22] van Albada M.E. (2007). Pulmonary Arterial Hypertension: Cardiovascular Effects of Pharmacological Intervention.

[23] Leberkühne L.J., Ploegstra M.J., Douwes J.M., Bartelds B., Roofthooft M.T.R., Hillege H.L., Berger R.M.F. (2017). Serially Measured Uric Acid Levels Predict Disease Severity and Outcome in Pediatric Pulmonary Arterial Hypertension. Am. J. Respir. Crit. Care Med..

[24] Watanabe T., Ishikawa M., Abe K., Ishikawa T., Imakiire S., Masaki K., Hosokawa K., Fukuuchi T., Kaneko K., Ohtsubo T. (2021). Increased Lung Uric Acid Deteriorates Pulmonary Arterial Hypertension. J. Am. Heart Assoc..

[25] Savale L., Akagi S., Tu L., Thuillet R., Bordenave J., Quatremare T., Cumont A., Phan C., Huertas A., Humbert M. (2018). Uric acid contributes to the progression of pulmonary hypertension in rodents and humans. Eur. Respir. J..

[26] Lewis R.A., Durrington C., Condliffe R., Kiely D.G. (2020). BNP/NT-proBNP in pulmonary arterial hypertension: Time for point-of-care testing?. Eur. Respir. Rev..

[27] Adachi S., Hirashiki A., Nakano Y., Shimazu S., Murohara T., Kondo T. (2014). Prognostic factors in pulmonary arterial hypertension with Dana Point group 1. Life Sci..

[28] Metkus T.S., Mullin C.J., Grandin E.W., Rame J.E., Tampakakis E., Hsu S., Kolb T.M., Damico R., Hassoun P.M., Kass D.A. (2016). Heart Rate Dependence of the Pulmonary Resistance x Compliance (RC) Time and Impact on Right Ventricular Load. PLoS ONE.

